# Longitudinal CT-Based Assessment of Muscle and Bone Changes After Liver Transplantation in Hepatitis B Patients with and Without Hepatocellular Carcinoma

**DOI:** 10.3390/diagnostics16091340

**Published:** 2026-04-29

**Authors:** Nurullah Dag, Sami Akbulut, Mahmut Sahin

**Affiliations:** 1 Department of Radiology, Inonu University Faculty of Medicine, 44280 Malatya, Türkiye; 2 Department of Surgery and Liver Transplantation, Inonu University Faculty of Medicine, 44280 Malatya, Türkiye; 3 Department of Biostatistics and Medical Informatics, Inonu University Faculty of Medicine, 44280 Malatya, Türkiye

**Keywords:** liver transplantation, hepatitis B virus, hepatocellular carcinoma, bone health, sarcopenia

## Abstract

**Background/Objectives:** Sarcopenia and impaired bone quality are increasingly recognized as important determinants of outcomes after liver transplantation (LT). However, longitudinal data describing early post-transplant musculoskeletal changes in patients with chronic hepatitis B virus (HBV) infection, particularly according to hepatocellular carcinoma (HCC) status, remain limited. **Aim**: To evaluate longitudinal changes in skeletal muscle mass and vertebral bone attenuation after LT in patients with chronic HBV infection and to assess the impact of concomitant HCC and clinical subgroups on these patterns. **Methods**: This retrospective, single-center study included 99 adult patients who underwent LT for chronic HBV infection (HBV alone, *n* = 59; HBV + HCC, *n* = 40) between January 2018 and December 2024. Contrast-enhanced abdominal computed tomography examinations obtained before transplantation and at approximately 6 (POD180) and 12 months (POD365) after transplantation were analyzed. Skeletal muscle was assessed using psoas muscle area (PMA) and psoas muscle index (PMI), while bone quality was evaluated using mean vertebral trabecular attenuation averaged across L1–4. Longitudinal changes were examined according to HCC status, sex, Child–Pugh class, and survival status. **Results**: Repeated-measures analyses of longitudinal changes demonstrated a significant decline in both PMA and PMI at POD180 and POD365 compared with pre-transplant values (PMA: *p* = 0.006; PMI: *p* = 0.009). These patterns were comparable between patients with HBV alone and those with HBV-related HCC, with no significant differences between groups (all *p* > 0.05). Male patients consistently exhibited higher PMA and PMI values than female patients across all assessed time points (both *p* < 0.001). In contrast, neither Child–Pugh class nor mortality status was associated with differences in PMA or PMI levels (all *p* > 0.05). L1–4 attenuation declined markedly by POD180 and remained below baseline at POD365 (*p* < 0.001). Although overall L1–4 values did not differ between disease groups (*p* = 0.109), the temporal pattern of L1–4 change differed according to survival status (*p* = 0.026), with a greater decline observed in non-survivors. **Conclusions**: In patients with chronic HBV undergoing LT, early post-transplant loss of skeletal muscle and vertebral bone attenuation is common and persists throughout the first year of follow-up. These changes occur similarly in patients with and without HCC. CT-based assessment of muscle and bone parameters, particularly L1–4 attenuation, may therefore support early post-transplant risk stratification.

## 1. Introduction

Chronic liver disease represents a major global health burden and is a systemic condition in which progressive hepatic dysfunction is accompanied by substantial disturbances in musculoskeletal integrity and bone metabolism, resulting from sustained liver injury. The most common etiologies of chronic liver disease worldwide include chronic viral hepatitis, autoimmune liver diseases, alcohol-associated liver disease, and metabolic dysfunction-associated steatotic liver disease—with chronic hepatitis B virus (HBV) infection accounting for a substantial proportion of cases, particularly in endemic regions [1,2]. In patients with chronic HBV infection, extrahepatic manifestations become progressively more prominent with advancing liver disease and may be further accentuated in the presence of hepatocellular carcinoma (HCC). Alterations in skeletal muscle mass and bone quality therefore carry substantial clinical relevance in this population, influencing transplant eligibility, perioperative risk, and long-term outcomes following liver transplantation (LT) [3,4].

The mechanisms underlying musculoskeletal deterioration in chronic HBV infection are multifactorial. Disruption of the liver–muscle axis, impaired ammonia detoxification, chronic systemic inflammation, hormonal imbalance, and altered energy metabolism collectively promote skeletal muscle catabolism and impair osteoblastic activity [5,6]. Beyond the effects of cirrhosis itself, HBV infection has been associated with reduced bone mineral density and an increased risk of osteoporosis [7]. Recent genetic and epidemiological data, including Mendelian randomization analyses, support a causal relationship between HBV infection and skeletal demineralization that extends beyond the severity of hepatic dysfunction alone [8].

The coexistence of HCC in patients with chronic HBV imposes an additional metabolic and inflammatory burden that may accelerate musculoskeletal decline. HCC is associated with tumor-driven metabolic reprogramming, heightened inflammatory signaling, and progressive protein catabolism, thereby contributing to the development of cancer-associated cachexia and sarcopenia [9,10]. Elevated circulating cytokines and chemokines, including interferon-gamma–induced protein 10, have been linked to muscle wasting in patients with HCC, highlighting the role of tumor-related inflammation in skeletal muscle loss [11]. In this setting, musculoskeletal deterioration is not limited to quantitative muscle loss, but also encompasses qualitative changes such as myosteatosis, which has emerged as an adverse prognostic factor in HCC independent of liver function and tumor stage [10,12]. Clinically, sarcopenia in patients with HCC has been associated with inferior responses to oncologic therapies, increased post-transplant complications, and reduced overall survival [13].

Within the context of LT, sarcopenia and impaired bone quality represent critical, yet potentially modifiable, determinants of outcome. Imaging-defined sarcopenia has consistently been linked to increased post-transplant mortality, prolonged hospitalization, and higher rates of infectious and metabolic complications [14,15,16]. The psoas muscle index (PMI), derived from cross-sectional imaging at the third lumbar vertebral (L3) level and normalized to patient height squared (m^2^), provides a practical surrogate marker of global skeletal muscle mass [4,14,17]. In parallel, reduced bone mineral density is highly prevalent among LT candidates, affecting up to 60% of patients, and has been shown to predict both post-transplant mortality and fragility fractures, particularly during the early post-transplant period when bone loss accelerates rapidly [1,18].

Opportunistic assessment using computed tomography (CT) offers a unique advantage for the simultaneous evaluation of muscle and bone health in this population. Routine abdominal CT examinations performed for HCC surveillance and transplant assessment allow for quantitative analysis of skeletal muscle and vertebral trabecular bone attenuation without additional imaging, radiation exposure, or cost [19,20]. CT-derived vertebral attenuation measured in Hounsfield unit (HU) at lumbar levels has demonstrated a strong correlation with dual-energy X-ray absorptiometry–derived bone mineral density and retains diagnostic accuracy even in contrast-enhanced studies, making it particularly suitable for opportunistic screening in patients undergoing serial imaging [20,21,22]. Moreover, CT enables longitudinal assessment of musculoskeletal changes, capturing dynamic alterations that may not be apparent with single time-point measurements. Given the limited availability of longitudinal comparative data describing musculoskeletal changes in chronic HBV patients undergoing LT, this study aimed to assess temporal changes in CT-derived PMI and vertebral trabecular attenuation and to compare these parameters between patients with and without HCC before and after LT.

## 2. Materials and Methods

### 2.1. Study Design and Population

This single-center, retrospective, observational study was conducted in accordance with established methodological standards for observational transplant research. Between January 2018 and December 2024, a total of 1825 LT procedures were performed at our center, of which 1451 involved adult recipients (≥18 years). Among these, 478 patients underwent LT due to chronic HBV infection, either without HCC (HBV alone) or with HBV-related HCC (HBV + HCC), and were considered potentially eligible. To be included in the study, patients were required to have at least 12 months of post-transplant imaging follow-up, with contrast-enhanced abdominal CT examinations available at all three predefined time points: pre-transplantation (PreLT), approximately 6 months (POD180), and 12 months (POD365) after LT. Of the 478 HBV-related adult recipients, 100 met these imaging follow-up criteria. Only CT examinations with adequate image quality for reliable assessment of skeletal muscle and vertebral trabecular attenuation were included. One patient was excluded due to inadequate CT image quality, resulting in a final study cohort of 99 patients. Thus, the final cohort consisted of patients with at least 12 months of follow-up and three serial CT examinations. Because inclusion required the availability of POD365 CT imaging, patients who died within the first postoperative year were not included in the final analysis.

Follow-up duration was defined as the time interval between the date of LT and the date of the last documented clinical assessment. For patients who were alive at the time of last follow-up, follow-up time was calculated from the LT to the most recent outpatient visit or recorded telephone contact. For patients who died during follow-up, follow-up time was calculated from the LT to the date of death. Survival status was determined based on institutional medical records and verified by direct contact when necessary.

### 2.2. CT Acquisition Protocol

All CT examinations were obtained as part of routine pre- and post-transplant surveillance using multidetector CT systems, including the Somatom Definition 256-slice scanner or equivalent clinical-grade scanners (Siemens Healthineers, Erlangen, Germany) and the Aquilion 64-slice scanner (Canon Medical Systems, Otawara, Japan). Image acquisition was performed according to a standardized multidetector CT protocol consistent with previously validated opportunistic CT musculoskeletal assessment methodologies [20]. All scans were acquired during the venous phase following intravenous contrast administration. CT acquisition parameters included a tube voltage of 120 kVp with automatic tube current modulation, a reconstruction matrix of 512 × 512, and a standard soft-tissue convolution kernel. For musculoskeletal assessment, images were reconstructed at a slice thickness of 1 mm to allow more detailed evaluation and reliable multiplanar reconstruction, particularly during first-year post-transplant follow-up and in patients undergoing HCC surveillance. Images were reviewed in both axial and sagittal planes to enable reliable quantitative assessment of skeletal muscle and vertebral trabecular bone.

### 2.3. Vertebral Trabecular Attenuation Measurements

Vertebral trabecular attenuation was assessed at the L1–4 levels on sagittal reformatted CT images using manually placed circular or ovoid ROIs within the central trabecular compartment, with exclusion of cortical bone, endplates, venous structures, and focal degenerative or sclerotic changes. Mean attenuation values (HU) were recorded for each vertebral level, and the average L1–4 value was used for subsequent analyses to improve robustness and reduce level-specific variability.

### 2.4. Psoas Muscle Measurements

Quantitative skeletal muscle assessment was performed at the level of the third lumbar vertebra (L3) on axial CT images, which is widely accepted as the anatomical reference standard for CT-based sarcopenia assessment, in accordance with established protocols. The right and left psoas muscles were manually delineated using freehand regions of interest at the axial slice where both transverse processes were clearly visualized. The cross-sectional areas of the bilateral psoas muscles were measured in square centimeters (cm^2^), and the sum of these measurements was defined as the psoas muscle area (PMA). To account for interindividual differences in body size, the PMI was calculated by normalizing PMA to the square of patient height (m^2^); PMI (cm^2^/m^2^) = PMA at L3 (cm^2^)/[height (m)]^2^, yielding PMI values expressed as cm^2^/m^2^, consistent with established CT-based sarcopenia assessment methods. This normalization approach is consistent with previously validated CT-based methods for sarcopenia assessment in patients with chronic liver disease and LT. Sarcopenia was defined using sex-specific cut-off values derived from the study cohort. The cut-off was determined as the 25th percentile (Q1) of preoperative PMI values separately for male and female patients [23,24,25]. Accordingly, sarcopenia was defined as PMI < 4.63 cm^2^/m^2^ in male and PMI < 3.69 cm^2^/m^2^ in female patients.

### 2.5. Image Analysis

All image analyses were performed on a dedicated workstation by radiologists with expertise in abdominal imaging who were blinded to clinical data and patient outcomes to minimize measurement and observer bias. Standardized measurement techniques were applied consistently across all examinations to minimize inter-scan and observer-related variability. All measurements were performed by radiologists who underwent a dedicated calibration session prior to the study to standardize ROI placement technique. Readers were blinded to clinical outcomes and group allocation. In cases of uncertainty regarding focal vertebral abnormalities, a consensus review was performed to ensure consistent exclusion criteria.

### 2.6. Immunosuppressive Protocol

Immunosuppression followed a standardized institutional protocol [26]. Induction therapy consisted of 500 mg intravenous methylprednisolone administered intraoperatively upon completion of hepatic artery anastomosis and achievement of hemostasis. High-dose corticosteroids were tapered over the first 10 postoperative days, followed by maintenance with a fixed daily dose of 5 mg dexamethasone until three months after transplantation. Corticosteroids were routinely discontinued at the end of the third postoperative month in the absence of autoimmune etiology or acute rejection requiring prolonged therapy.

Mycophenolate mofetil was initiated at a dose of 500–1000 mg twice daily in the early postoperative period and was generally discontinued at six months. Tacrolimus therapy was initiated on postoperative day 3, with target trough levels maintained at 8–10 ng/mL during the first postoperative year. In patients with perioperative renal dysfunction, tacrolimus initiation was postponed until renal function improved, typically during the first postoperative week. In such cases, basiliximab induction was administered beginning on postoperative day 1 and repeated weekly as needed. If renal function failed to recover and serum creatinine levels remained above 2 mg/dL, tacrolimus-free regimens or low-dose, sustained-release tacrolimus combined with MMF were employed. Importantly, none of the patients included in the present study had hepatorenal syndrome or perioperative renal impairment.

In recipients transplanted for HCC, an mTOR inhibitor (everolimus) was typically introduced after completion of early wound healing, approximately one month post-transplantation. Everolimus trough levels were maintained between 3–5 ng/mL. Regarding factors potentially influencing bone metabolism, no patient required prolonged corticosteroid therapy for early rejection. Routine vitamin D supplementation was not implemented either before or after transplantation. Although calcium–vitamin D combination therapy has been prescribed more recently in selected patients, adherence has been limited due to gastrointestinal intolerance. None of the patients included in this study received routine vitamin D supplementation or bisphosphonate therapy.

### 2.7. Antiviral Therapy Protocol

In patients with chronic HBV infection, antiviral therapy was initiated according to established clinical criteria. In the presence of cirrhosis and detectable HBV-DNA, treatment with a potent nucleos(t)ide analogue—tenofovir disoproxil fumarate, tenofovir alafenamide, or entecavir—was initiated irrespective of viral load level.

In non-cirrhotic patients, antiviral therapy was guided by HBV-DNA level and fibrosis assessment. When HBV-DNA exceeded 2000 IU/L, treatment was initiated in patients aged >40 years or in those with evidence of significant fibrosis, defined as FIB-4 score >1.45, transient elastography (FibroScan) >8 kPa, shear wave elastography >3.4 m/s, or magnetic resonance elastography >3.2 kPa. In cases without noninvasive evidence of fibrosis, liver biopsy was performed; antiviral therapy was initiated if histological activity index (HAI) was >4 or fibrosis stage was ≥F2. In patients with HBV-related HCC, antiviral therapy with a potent nucleos(t)ide analogue was initiated if HBV-DNA was detectable.

### 2.8. Study Protocol, Ethical Considerations, and Funding

This study was conducted in accordance with the ethical principles of the Declaration of Helsinki (1964) and its subsequent amendments, and complied with all applicable institutional and national regulations governing research involving human participants. Ethical approval was obtained from the Inonu University Institutional Review Board (IRB) for Non-Interventional Studies (Approval Date: 30 December 2025; Approval number: 9118). As the study had a retrospective design involving review of medical records and re-evaluation of archived radiological images, the requirement for individual informed consent was waived. The study received financial support from the Inonu University Scientific Research Projects Coordination Unit (Grant ID: TSA-2026-4616). The study was designed, conducted, analyzed, and reported in accordance with the STROBE (Strengthening the Reporting of Observational Studies in Epidemiology) guidelines to enhance transparency, reproducibility, and methodological rigor [27].

### 2.9. Statistical Analysis

Statistical analyses were performed using IBM SPSS Statistics for Windows, version 25.0 (IBM Corp., Armonk, NY, USA). Graphs were generated using GraphPad Prism version 10.6.1 for Windows (GraphPad Software, Boston, MA, USA). Continuous variables were summarized as mean ± standard deviation or median (interquartile range), whereas categorical variables were presented as counts and percentages. The normality of distribution for continuous variables was assessed using the Shapiro–Wilk test. Homogeneity of variances was evaluated using Levene’s test for comparisons between independent groups. For comparisons between two groups, Student’s *t*-test was used for normally distributed variables, whereas the Mann–Whitney U test was used for non-normally distributed variables. For comparisons involving more than two groups, one-way analysis of variance (ANOVA) was used for normally distributed variables, followed by Bonferroni-adjusted post hoc analysis when appropriate. Categorical variables were compared using the chi-square test.

Repeated-measures analysis of variance (repeated-measures ANOVA) was initially used to evaluate measurements obtained from the same individuals at different time points. The sphericity assumption was assessed using Mauchly’s test, and the Greenhouse–Geisser correction was applied when this assumption was violated. Time effect, group effect, and time × group interaction were analyzed separately. For statistically significant time effects, Bonferroni-adjusted pairwise comparisons were performed. Both within-subject changes over time and between-group differences were evaluated within the same analytical framework for sex, Child–Pugh class, HBV and HBV + HCC groups, and clinical outcome (Survivors vs. Non-survivors). Effect size in repeated-measures analyses was reported as partial eta squared (η^2^).

To account for potential confounding arising from baseline clinical differences between groups, multivariable linear mixed-effects models were additionally constructed for longitudinal musculoskeletal parameters (L1–4 attenuation, PMA, and PMI). In these models, time, group, and time-by-group interaction were specified as fixed effects. Age, MELD score, sex, INR, albumin level, and platelet count were included as covariates. Patient ID was specified as the subject variable to account for within-subject correlations across repeated measurements, and a compound symmetry covariance structure was applied. In exploratory analyses, a three-way interaction term (Time × Group × Sex) was also evaluated to assess potential sex-specific modification of longitudinal trajectories. A *p* value < 0.05 was considered statistically significant for all analyses.

## 3. Results

### 3.1. Baseline Demographic, Clinical, and Laboratory Characteristics

Table 1 presents the baseline demographic, clinical, and laboratory characteristics of patients with HBV (*n* = 59) and HBV + HCC (*n* = 40). Patients in the HBV + HCC group were significantly older than those with HBV alone (*p* = 0.001). MELD scores were higher in the HBV Alone group (*p* = 0.002), whereas pre-transplant AFP levels were markedly higher in the HBV + HCC group (*p* = 0.001). Significant between-group differences were also observed for total bilirubin (*p* = 0.008), GGT (*p* = 0.016), INR (*p* < 0.001), albumin (*p* = 0.025), BUN (*p* = 0.024), calcium (*p* = 0.006), white blood cell count (*p* = 0.004), hemoglobin (*p* = 0.003), platelet count (*p* = 0.001), total cholesterol (*p* = 0.012), LDL cholesterol (*p* = 0.008), and triglyceride levels (*p* = 0.014). In contrast, anthropometric measures (height, weight, BMI, BSA), ALP, sodium, creatinine, uric acid, phosphorus, HDL cholesterol, follow-up duration, and baseline as well as follow-up PMA, PMI, and L1–4 measurements did not differ significantly between groups.

Table 2 summarizes the distribution of categorical variables. Male sex was significantly more prevalent in the HBV + HCC group than in the HBV Alone group (97.5% vs. 76.3%, *p* = 0.004). Child–Pugh class distribution also differed significantly between groups (*p* < 0.001), with a higher proportion of Child–Pugh A patients in the HBV + HCC group and a greater proportion of Child–Pugh B and C patients in the HBV Alone group. In contrast, no significant between-group differences were observed in blood group distribution, LT type, or the prevalence of sarcopenia based on the PMI cut-off at PreLT, POD180, and POD365. Survival outcome also differed significantly between groups, with a higher proportion of non-survivors in the HBV + HCC group than in the HBV Alone group (27.5% vs. 6.8%, *p* = 0.005). As inclusion required the availability of POD365 CT imaging, all deaths occurred beyond the first postoperative year. Follow-up duration also differed significantly according to survival status, with survivors having a longer follow-up duration than non-survivors (median, 1987 [1310–2443] days vs. 896 [607–1513] days, respectively; *p* = 0.002).

### 3.2. Bivariate Comparisons of PMA Across Clinical Subgroups at Each Time Point

**Figure 1:**
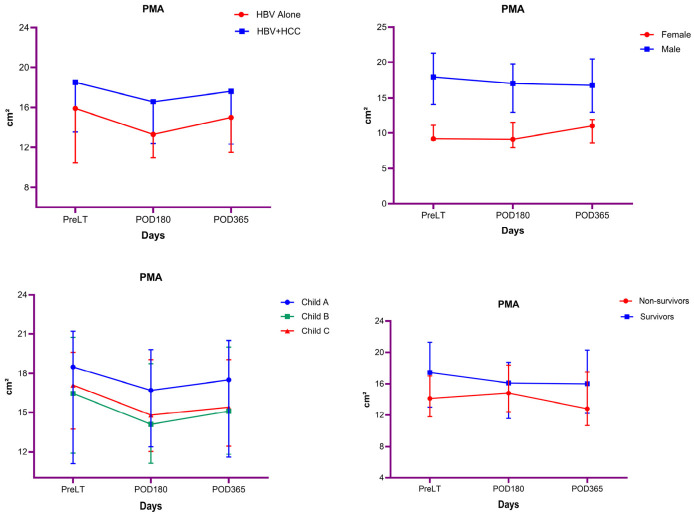
Longitudinal changes in PMA across clinical subgroups at PreLT, POD180, and POD365. Values are expressed as median (interquartile range).

Supplementary Table S1 shows the bivariate comparisons of PMA values at PreLT, POD180, and POD365 according to clinical subgroups. PMA values did not differ significantly between HBV Alone and HBV + HCC groups at any time point (all *p* > 0.05). In contrast, male patients had significantly higher PMA values than female patients across all time points (PreLT: *p* < 0.001; POD180: *p* < 0.001; POD365: *p* < 0.001). No significant differences in PMA were observed across Child–Pugh classes or according to survival status in the bivariate analyses (all *p* > 0.05). These findings are also illustrated in Figure 1.

### 3.3. Longitudinal Changes in PMA According to Clinical Subgroups

Table 3 shows the results of repeated-measures ANOVA evaluating longitudinal changes in PMA. In the analyses comparing groups, PMA showed a significant change over time (*p* = 0.006; partial η^2^ = 0.054). Pairwise comparisons revealed significant reductions in PMA at POD180 and POD365 compared with PreLT (PreLT–POD180: *p* = 0.001; PreLT–POD365: *p* = 0.037), while the difference between POD180 and POD365 was not significant. The interaction between time and group was not statistically significant, indicating comparable temporal trajectories of PMA in both groups. Moreover, overall mean PMA levels did not differ significantly between groups.

In the analyses comparing female and male patients, PMA did not show a significant change over time. Pairwise comparisons demonstrated no significant differences in PMA between PreLT and POD180, PreLT and POD365, or POD180 and POD365. The interaction between time and sex was not statistically significant, indicating comparable temporal trajectories of PMA in female and male patients. In contrast, overall mean PMA levels differed significantly between sexes, with male patients exhibiting higher PMA values than female patients across all time points (*p* < 0.001).

According to Child–Pugh class, PMA showed a significant change over time (*p* = 0.015; partial η^2^ = 0.046). Pairwise comparisons demonstrated a significant reduction in PMA at POD180 compared with the PreLT period (*p* = 0.010), whereas no significant differences were observed between PreLT and POD365 or between POD180 and POD365. The interaction between time and Child–Pugh class (A, B, and C) was not statistically significant, indicating comparable temporal trajectories of PMA across all Child–Pugh classes. Moreover, overall mean PMA levels did not differ significantly among Child–Pugh A, B, and C patients.

According to survival status, PMA did not show a significant change over time. Pairwise comparisons revealed no significant differences in PMA between PreLT and POD180, PreLT and POD365, or POD180 and POD365. The interaction between time and mortality status was not statistically significant, indicating comparable temporal trajectories of PMA irrespective of mortality status. Moreover, overall mean PMA levels did not differ significantly between survivors and non-survivors.

### 3.4. Bivariate Comparisons of PMI Across Clinical Subgroups at Each Time Point

**Figure 2:**
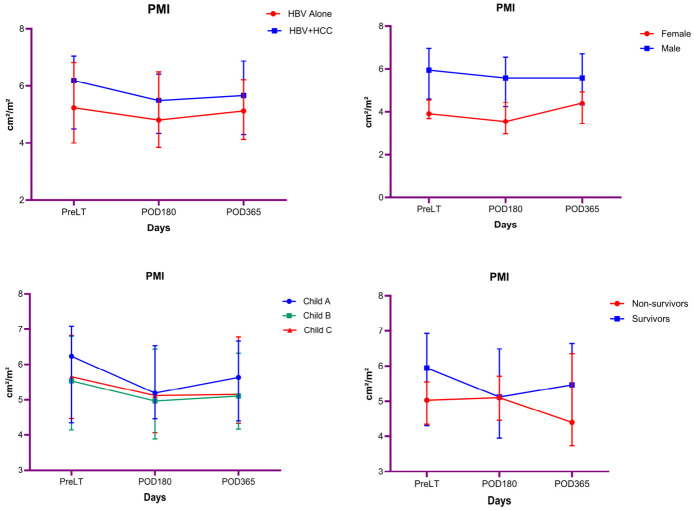
Longitudinal changes in PMI across clinical subgroups at PreLT, POD180, and POD365. Values are expressed as median (interquartile range).

Supplementary Table S2 presents the bivariate comparisons of PMI values at PreLT, POD180, and POD365 according to clinical subgroups. PMI values did not differ significantly between the HBV and HBV + HCC groups at any time point (all *p* > 0.05). In contrast, male patients had significantly higher PMI values than female patients across all time points (PreLT: *p* < 0.001; POD180: *p* < 0.001; POD365: *p* < 0.001). No significant differences in PMI were observed across Child–Pugh classes or according to survival status in the bivariate analyses (all *p* > 0.05). These findings are also demonstrated in Figure 2.

### 3.5. Longitudinal Changes in PMI According to Clinical Subgroups

Table 4 shows the results of repeated-measures ANOVA evaluating longitudinal changes in PMI. When comparing groups, PMI showed a significant change over time (*p* = 0.009; partial η^2^ = 0.051). Pairwise comparisons revealed significant reductions in PMI at POD180 and POD365 compared with PreLT (PreLT–POD180: *p* = 0.001; PreLT–POD365: *p* = 0.047), whereas the difference between POD180 and POD365 was not significant. The interaction between time and group was not statistically significant, indicating comparable temporal trajectories of PMI in both groups. Moreover, overall mean PMI levels did not differ significantly between groups.

According to sex, PMI did not show a significant change over time. Pairwise comparisons demonstrated a significant difference between PreLT and POD180 (*p* = 0.040), whereas no significant differences were observed between PreLT and POD365 or between POD180 and POD365. The interaction between time and sex was not statistically significant, indicating comparable temporal trajectories of PMI in female and male patients. In contrast, overall mean PMI levels differed significantly between sexes, with male patients exhibiting higher PMI values than female patients across all time points (*p* < 0.001; partial η^2^ = 0.151).

According to mortality status, PMI did not show a significant change over time. Pairwise comparisons revealed no significant differences between PreLT and POD180, PreLT and POD365, or POD180 and POD365. The interaction between time and mortality status was not statistically significant, indicating comparable temporal trajectories of PMI irrespective of mortality status. Moreover, overall mean PMI levels did not differ significantly between survivors and non-survivors.

According to Child–Pugh class (A, B, and C), PMI showed a significant change over time (*p* = 0.017; partial η^2^ = 0.044). Pairwise comparisons demonstrated a significant reduction in PMI from PreLT to POD180 (*p* = 0.009), whereas the differences between PreLT and POD365 and between POD180 and POD365 were not statistically significant. The interaction between time and Child–Pugh class was not statistically significant, indicating similar temporal patterns of PMI across Child–Pugh classes. In addition, overall mean PMI levels did not differ significantly between Child–Pugh classes.

### 3.6. Bivariate Comparisons of Vertebral Trabecular Attenuation Across Clinical Subgroups at Each Time Point

**Figure 3:**
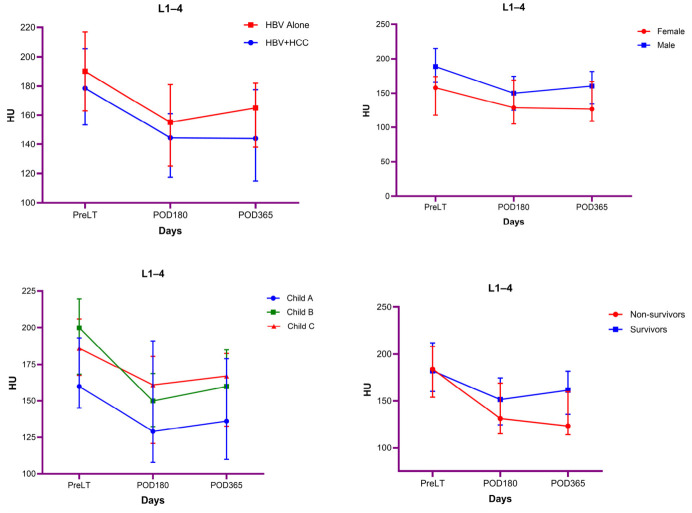
Longitudinal changes in vertebral trabecular attenuation across clinical subgroups at PreLT, POD180, and POD365. Values are expressed as median (interquartile range).

Supplementary Table S3 presents the bivariate comparisons of L1–4 attenuation values at PreLT, POD180, and POD365 across clinical subgroups. L1–4 attenuation did not differ significantly between the HBV Alone and HBV + HCC groups at any time point (all *p* > 0.05). Male patients had significantly higher L1–4 attenuation values than female patients at PreLT (*p* = 0.018) and POD365 (*p* = 0.032), whereas no significant sex-based difference was observed at POD180 (*p* = 0.340). Differences across Child–Pugh classes were significant at PreLT (*p* = 0.048) but not at POD180 or POD365 (both *p* > 0.05). No significant differences were observed according to survival status at any time point (all *p* > 0.05). These findings are also shown in Figure 3.

### 3.7. Longitudinal Changes in Vertebral Trabecular Attenuation According to Clinical Subgroups

Table 5 shows the results of repeated-measures ANOVA evaluating longitudinal changes in L1–4 attenuation. When comparing groups, L1–4 measurements changed significantly over time (*p* < 0.001; partial η^2^ = 0.443). Pairwise comparisons demonstrated significant decreases in L1–4 values at POD180 and POD365 compared with PreLT (both *p* < 0.001), whereas the difference between POD180 and POD365 did not reach statistical significance. The interaction between time and group was not statistically significant, indicating similar temporal patterns of L1–4 change in both groups. Overall mean L1–4 levels did not differ significantly between groups.

According to sex, L1–4 values demonstrated a significant change over time (*p* < 0.001; partial η^2^ = 0.246). Pairwise comparisons revealed significant reductions in L1–4 at POD180 and POD365 compared with PreLT (both *p* < 0.001), while no significant difference was observed between POD180 and POD365. The time-by-sex interaction was not statistically significant, indicating that the temporal trajectory of L1–4 was comparable between male and female patients. Overall mean L1–4 values were significantly higher in male patients than in female patients (*p* = 0.047).

According to Child–Pugh class, L1–4 measurements changed significantly over time (*p* < 0.001; partial η^2^ = 0.388). Pairwise comparisons revealed significant decreases in L1–4 values at POD180 and POD365 compared with PreLT (both *p* < 0.001), whereas the difference between POD180 and POD365 was not statistically significant. The interaction between time and Child–Pugh class (A, B, and C) was not statistically significant, indicating that the temporal change in L1–4 values was similar across all Child–Pugh groups. Overall mean L1–4 levels did not differ significantly across Child–Pugh classes.

According to survival status, L1–4 changed significantly over time (*p* < 0.001; partial η^2^ = 0.374). Pairwise comparisons demonstrated significant reductions in L1–4 values at POD180 and POD365 compared with PreLT (both *p* < 0.001), while the difference between POD180 and POD365 was not statistically significant. The interaction between time and mortality status was statistically significant (*p* = 0.026), indicating that the temporal pattern of L1–4 change differed between survivors and non-survivors. However, overall mean L1–4 levels did not differ significantly according to survival status.

### 3.8. Multivariable-Adjusted Longitudinal Analysis

To account for potential confounding related to measured baseline clinical differences between groups, multivariable linear mixed-effects models were constructed for each longitudinal musculoskeletal parameter (L1–4 attenuation, PMA, and PMI). In each model, time, group (HBV Alone vs. HBV + HCC), and the time-by-group interaction were included as fixed effects. Age, MELD score, sex, INR, albumin level, and platelet count were entered as covariates. Repeated measurements were clustered within patients, and a compound symmetry covariance structure was specified to account for within-subject correlation across time. In exploratory analyses, a three-way interaction term (time × group × sex) was additionally evaluated to assess sex-specific modification of longitudinal trajectories. After multivariable adjustment, the time-by-group interaction remained non-significant for L1–4 attenuation (*p* = 0.559), PMA (*p* = 0.229), and PMI (*p* = 0.296), indicating that longitudinal changes in musculoskeletal parameters were not significantly modified by HCC status. A significant main effect of time persisted across models, whereas no independent main effect of group was observed.

## 4. Discussion

In this longitudinal CT-based study of patients undergoing LT for chronic HBV infection, musculoskeletal parameters exhibited a consistent temporal pattern during the first post-transplant year. Both PMI and lumbar vertebral trabecular attenuation declined markedly by POD180, with only limited recovery by POD365, without recovery to pre-transplant levels. Importantly, these longitudinal changes were comparable between patients with HBV alone and those with HBV-related HCC, as no significant group-by-time interactions were observed for PMI or L1–4 attenuation. Despite baseline clinical differences between groups, including older age, lower MELD scores, and higher platelet counts in the HBV + HCC cohort, HCC status did not translate into differential post-transplant musculoskeletal trajectories. The longitudinal changes in PMI and vertebral trabecular attenuation were not modified by HCC status, whereas vertebral trabecular attenuation showed a significant time × survival interaction, indicating different temporal trajectories according to survival outcome. Collectively, these findings indicate that early post-transplant musculoskeletal deterioration in HBV patients follows a shared temporal course that appears independent of HCC status but closely linked to patient-level and LT-related factors.

Chronic HBV infection constitutes an important clinical context for interpreting the skeletal changes observed in our cohort. Patients with HBV-related cirrhosis frequently exhibit reduced bone mineral density and an increased prevalence of osteoporosis, particularly in advanced stages of liver disease [28]. In the present study, vertebral trabecular attenuation declined most prominently within the first 6 months after LT and demonstrated only partial recovery by 12 months, without reaching pre-transplant levels. This pattern suggests that early post-transplant bone loss reflects the combined influence of pre-existing HBV-related metabolic bone disease and transplantation-related factors, including corticosteroid exposure, persistent alterations in calcium and vitamin D metabolism, systemic inflammation, and reduced mobility during the early recovery period. Although improvement in liver function after transplantation may alleviate some contributors to bone loss, our findings indicate that skeletal recovery remains incomplete during the first post-transplant year. In addition, chronic HBV infection itself has been linked to increased bone turnover and skeletal demineralization independent of liver disease severity, as supported by genetic and observational evidence [8]. Antiviral therapy may further influence bone trajectories, with several studies reporting greater bone mineral density decline in patients treated with tenofovir disoproxil fumarate compared with entecavir, although results across cohorts remain heterogeneous [29,30]. Taken together, these observations underscore the multifactorial nature of bone loss in chronic HBV infection and highlight the need for longitudinal skeletal assessment and early bone-protective strategies after LT, irrespective of HCC status. The divergent longitudinal patterns of vertebral trabecular attenuation according to mortality status further suggest that early post-transplant bone loss may reflect broader systemic vulnerability rather than isolated skeletal pathology.

The finding that post-transplant PMI changes over time were similar in patients with and without HCC in our cohort should not be interpreted as being in direct contradiction with the existing literature, because the available studies have largely addressed a different clinical question. Beumer et al. [31], in a multicenter study of patients transplanted for HCC beyond the Milan criteria, showed that lower pre-transplant skeletal muscle mass and sarcopenia were associated with inferior long-term survival; however, that study was restricted to an HCC-only population and did not compare HCC and non-HCC recipients. Li et al. [32] similarly evaluated only HCC recipients undergoing LT and examined multiple pre-transplant CT-derived body composition parameters, including SMI, PMI, and skeletal muscle density; in that analysis, impaired muscle quality, particularly lower skeletal muscle density/myosteatosis, showed the strongest association with post-transplant survival after adjustment for covariates. In the same disease setting, Lu et al. [33] reported that, among HCC recipients, pre-transplant myosteatosis aggravated the adverse prognostic effect of sarcopenia, while excessive post-transplant muscle loss was independently associated with worse overall and recurrence-free survival in non-sarcopenic recipients. Thus, these studies establish the prognostic relevance of muscle depletion and impaired muscle quality within HCC transplant populations, rather than demonstrating that the mere presence of HCC independently results in lower muscle mass compared with non-HCC recipients. This broader interpretation is also consistent with meta-analytic evidence showing that myosteatosis is associated with worse survival in HCC [10] and that sarcopenia is associated with increased post-transplant mortality in LT populations overall [13]. To our knowledge, direct comparative studies evaluating sarcopenia across HCC and non-HCC chronic liver disease populations remain very limited, and studies closely matching the present etiology-matched, longitudinal transplant design are almost absent.

In contrast to those prognostic studies, our study specifically examined whether longitudinal post-transplant muscle trajectories differed according to HCC status within a homogeneous HBV transplant cohort and showed that post-transplant PMI changes over time were similar in patients with and without HCC. In a comparative study more closely aligned with this question, D’Arcangelo et al. [34] reported that although sarcopenia was common in cirrhotic patients with and without HCC undergoing LT, its prognostic impact was more evident in patients with advanced hepatic decompensation and less pronounced overall in the HCC subgroup, with a signal mainly in those beyond Milan criteria. In addition, outside the transplant setting, Cimsit et al. [35] reported higher rates of sarcopenia and sarcopenic obesity in patients with HCC than in patients with chronic hepatitis B, although no significant difference was observed between the HCC and cirrhosis groups. Taken together, these observations suggest that the relationship between HCC and muscle depletion is highly context dependent and may be shaped by disease stage, transplant selection, and timing, which provides a plausible framework for the neutral PMI findings observed in the present cohort.

In parallel with the longitudinal muscle-related findings, vertebral trabecular attenuation followed a similar longitudinal pattern in patients with and without HCC in our cohort. This should not be interpreted as being in direct conflict with the existing bone-related literature, because the available studies have largely focused on HCC populations alone rather than directly comparing HCC and non-HCC groups. Meister et al. [36], in a European cohort of patients undergoing partial hepatectomy for HCC, reported that osteopenia was independently associated with inferior overall survival and worse disease-free survival, thereby highlighting the prognostic relevance of reduced bone mineral density within HCC surgical populations. Tibbetts et al. [37], in a retrospective cohort of patients younger than 65 years with HCC, demonstrated a high prevalence of low bone mineral density based on opportunistic CT attenuation, supporting the finding that bone demineralization is common even in relatively younger HCC patients. Likewise, Toshima et al. [38] showed that preoperative osteopenia was an independent risk factor for post-transplant mortality in patients undergoing living donor liver transplantation for HCC. Taken together, these studies support the clinical importance of low bone mineral density in HCC, but they do not directly demonstrate that the mere presence of HCC results in lower vertebral attenuation compared with non-HCC chronic liver disease populations. In contrast, our study addressed a different question, namely whether longitudinal vertebral attenuation trajectories differed between HBV patients with and without HCC, and found similar temporal patterns in the two groups. The absence of an HCC-related difference in vertebral attenuation in our cohort may therefore reflect population-specific characteristics as well as the timing of transplantation. Although HCC is associated with inflammatory and metabolic alterations known to negatively affect bone metabolism [7,39], these processes are likely progressive and may require prolonged exposure to produce measurable skeletal divergence. Early transplantation, particularly in settings with shorter waiting times, may therefore limit the cumulative impact of tumor-related catabolic stress on bone tissue, resulting in comparable attenuation trajectories between patients with and without HCC.

The predominance of LDLT in our cohort may have contributed to the absence of distinct HCC-related effects on muscle and bone parameters. LDLT offers an opportunity to reduce waitlist burden and waitlist mortality compared with deceased donor pathways [40,41]. In this context, transplantation may also occur earlier in the disease course, potentially limiting cumulative exposure to ongoing tumor-related catabolic drive [42]. In our HBV + HCC subgroup, 42.5% (*n* = 17) of patients were within the Milan criteria, whereas 57.5% (*n* = 23) were beyond the Milan criteria. However, the Milan criteria are based primarily on tumor size and number and do not directly capture tumor biology [43]. Accordingly, several LDLT-oriented selection frameworks have attempted to address this limitation by incorporating additional biological features, such as alpha-fetoprotein (AFP), des-gamma-carboxyprothrombin (DCP), tumor differentiation, and cancer-related symptoms, rather than relying solely on morphologic burden [43,44]. In this context, selected patients beyond the Milan criteria may still undergo LT without necessarily harboring biologically aggressive disease. This may partly explain why the systemic effects of HCC appeared less pronounced in our cohort. Consistent with this interpretation, previous studies suggest that the clinical impact of sarcopenia is most evident in patients with advanced hepatic decompensation, whereas its effect is less apparent in compensated disease and in selected HCC transplant populations [34,45]. Together, these factors provide a plausible explanation for the similar musculoskeletal patterns observed in patients with and without HCC in the present study, although this issue should be further clarified in prospective studies incorporating HCC-specific biological features.

An additional point that should be considered when interpreting these findings is the fundamental difference in transplant indication between the two groups. Patients in the HBV Alone group generally underwent transplantation because of more advanced liver dysfunction, whereas those in the HBV + HCC group were transplanted primarily for oncologic indications and may have had relatively better preserved baseline liver function. Accordingly, although post-transplant musculoskeletal trajectories were similar between groups after multivariable adjustment, these comparable patterns may have reflected different underlying pre-transplant drivers, including tumor-related effects in the HBV + HCC group and more advanced hepatic dysfunction in the HBV Alone group. This possibility should be acknowledged as an important interpretive limitation and warrants further evaluation in prospective studies with more detailed biological and functional stratification.

Taken together, these findings underscore the early post-transplant period as a particularly vulnerable phase for musculoskeletal deterioration in patients with chronic HBV infection, regardless of HCC status. Routine abdominal CT examinations obtained for transplant evaluation and follow-up provide an opportunity to assess both skeletal muscle mass and vertebral trabecular attenuation longitudinally without additional imaging or radiation exposure [16,37,38]. The ability to identify patients demonstrating early declines in these parameters may support closer clinical monitoring and inform timely nutritional, rehabilitative, and bone-focused interventions aimed at mitigating longer-term functional impairment after LT.

## 5. Limitations

This study has several limitations. First, its retrospective single-center design and relatively small sample size may limit the generalizability of the findings and reduce the ability to detect subtle differences between subgroups. Second, although measurements were performed using a standardized protocol by experienced radiologists, formal inter- and intra-observer reproducibility analyses were not conducted, which may limit the assessment of measurement robustness. Third, a notable limitation is the marked male predominance within the HBV + HCC subgroup (39/40 patients, 97.5% male). Although this distribution is consistent with the known epidemiology of HBV-related HCC, it restricts sex-specific interpretation. In multivariable linear mixed-effects models adjusted for age, MELD score, INR, albumin level, and platelet count, sex was included as a covariate. In addition, a three-way interaction term (Time × Group × Sex) was tested for PMI and was not statistically significant (F = 1.237, *p* = 0.293). However, the extremely small number of female patients in the HBV + HCC subgroup substantially limits statistical power to detect potential sex-specific longitudinal differences. Therefore, the findings in this subgroup should primarily be interpreted as reflecting male patient trajectories, and caution is warranted when generalizing these results to female patients. Finally, functional measures of muscle performance and biochemical markers of bone metabolism were not available, which might have provided additional insight into the clinical relevance of the imaging findings. Future prospective, multicenter studies with larger and more sex-balanced cohorts, as well as integrated functional and laboratory assessments, are warranted to confirm and extend these results.

## 6. Conclusions

In LT recipients with chronic HBV infection, both psoas muscle indices and vertebral trabecular attenuation decline predominantly within the first 6 months after transplantation, with only partial recovery by 12 months that does not reach pre-transplant levels. Musculoskeletal trajectories are similar in patients with and without HCC, suggesting that early post-transplant changes are largely driven by transplantation-related factors rather than malignancy status. The association between longitudinal vertebral attenuation patterns and mortality highlights the potential value of opportunistic CT-based musculoskeletal assessment for early risk stratification after LT.

## Figures and Tables

**Table 1:** Baseline demographic, clinical, and laboratory characteristics of HBV alone versus HBV + HCC groups.

Variable	HBV Alone		HBV + HCC		*p*
	Mean ± SD	Median (Q1–Q3)	Mean ± SD	Median (Q1–Q3)	
Age	50 ± 10	52 (45–57)	57 ± 9	57 (53–62)	0.001 ^t^
Height	171 ± 10	170 (165–178)	172 ± 6	171 (170–176)	0.386 ^t^
Weight	79 ± 14	80 (69–90)	78 ± 13	77 (67–85)	0.701 ^t^
BMI	27.1 ± 5.0	27 (24–31)	26.2 ± 4.4	25 (23–30)	0.352 ^t^
BSA	1.9 ± 0.2	1.9 (1.8–2.1)	1.9 ± 0.2	1.9 (1.8–2.0)	0.896 ^t^
MELD Score	18 ± 7.0	16 (14–22)	14 ± 7.3	13 (9–16)	0.002 ^u^
PreLT AFP	21.7 ± 50	5.6 (3.0–20.8)	347 ± 1645	11.5 (6.0–65.5)	0.001 ^u^
Total Bilirubin	4.0 ± 5.2	2.5 (1.3–3.7)	2.8 ± 3.8	1.4 (1.0–2.1)	0.008 ^u^
ALP	119 ± 51	111 (83–158)	133 ± 54	126 (81–162)	0.255 ^u^
GGT	88 ± 89	50 (33–106)	115 ± 84	80 (46–166)	0.016 ^u^
INR	1.5 ± 0.5	1.4 (1.3–1.7)	1.3 ± 0.2	1.2 (1.1–1.4)	<0.001 ^u^
Albumin	2.9 ± 0.6	2.9 (2.4–3.3)	3.2 ± 0.7	3.2 (2.7–3.7)	0.025 ^t^
Na	136 ± 4	136 (133–139)	133 ± 22	137 (134–139)	0.076 ^u^
BUN	14.8 ± 9.6	12.6 (10.7–16)	16.6 ± 7.5	15 (12–21)	0.024 ^u^
Creatinine	0.9 ± 0.5	0.8 (0.7–0.9)	0.9 ± 0.2	0.9 (0.7–1)	0.091 ^u^
Uric Acid	4.5 ± 1.8	4.2 (3.4–5.0)	4.6 ± 1.4	4.5 (3.5–5)	0.424 ^u^
Calcium	8.4 ± 0.9	8.4 (7.9–8.8)	8.8 ± 0.8	8.8 (8.3–9)	0.006 ^u^
Phosphorus	3.1 ± 0.8	3.1 (2.6–3.6)	3.0 ± 0.7	2.9 (2.6–3.5)	0.566 ^u^
WBC	4.8 ± 2.1	4.5 (3.3–5.8)	5.8 ± 1.9	5.9 (4.7–7.2)	0.004 ^u^
Hemoglobin	11.8 ± 2.1	11.8 (9.8–13.6)	13 ± 2.3	14 (12–15)	0.003 ^t^
Platelets	86 ± 44	76 (58–106)	123 ± 61	111 (71–164)	0.001 ^u^
Total Cholesterol	126 ± 41	127 (98–149)	147 ± 37	144 (120–171)	0.012 ^t^
HDL	38 ± 15	38 (28–49)	40 ± 14	39 (33–51)	0.437 ^t^
LDL	71 ± 29	69 (49–91)	92 ± 41	88 (69–115)	0.008 ^u^
Triglyceride	84 ± 42	73 (57–106)	101 ± 38	91 (73–132)	0.014 ^u^
L1–4 (PreLT)	190 ± 45	190 (163–217)	181 ± 38	179 (154–205)	0.289 ^t^
L1–4 (POD180)	158 ± 43	155 (125–181)	142 ± 38	145 (118–161)	0.072 ^u^
L1–4 (POD365)	163 ± 41	165 (138–182)	150 ± 43	144 (116–177)	0.088 ^u^
PMA (PreLT)	16.2 ± 5.8	16 (11–20)	17.5 ± 4.4	19 (14–21)	0.226 ^t^
PMA (POD180)	15.5 ± 5.9	13 (11–19)	15.9 ± 4.7	17 (12–19)	0.720 ^t^
PMA (POD365)	15.3 ± 5.5	15 (12–18)	16.4 ± 5.5	18 (12–21)	0.342 ^t^
PMI (PreLT)	5.5 ± 1.8	5.2 (4.0–6.8)	5.9 ± 1.4	6.2 (4.5–7.0)	0.277 ^t^
PMI (POD180)	5.2 ± 1.8	4.8 (3.8–6.5)	5.3 ± 1.6	5.5 (4.4–6.4)	0.749 ^t^
PMI (POD365)	5.2 ± 1.7	5.1 (4–6.2)	5.5 ± 1.8	5.7 (4.3–6.8)	0.409 ^t^
Follow up (days)	1781 ± 676	1897 (1303–2297)	1852 ± 1232	1726 (760–2503)	0.770 ^u^

Abbreviations: HBV, hepatitis B virus; HCC, hepatocellular carcinoma; SD, standard deviation; Q1–Q3, first to third quartile; BMI, body mass index; BSA, body surface area; MELD, Model for End-stage Liver Disease; PreLT, pre-liver transplantation; AFP, alpha-fetoprotein; ALP, alkaline phosphatase; GGT, gamma-glutamyl transferase; INR, international normalized ratio; Na, sodium; BUN, blood urea nitrogen; WBC, white blood cell; HDL, high-density lipoprotein; LDL, low-density lipoprotein; L1–4, lumbar vertebrae 1–4; PMA, psoas muscle area; PMI, psoas muscle index; POD180, postoperative day 180; POD365, postoperative day 365. Superscript t indicates independent samples *t*-test; superscript u indicates Mann–Whitney U test.

**Table 2:** Distribution of categorical variables in HBV Alone versus HBV + HCC groups.

Variables	Category	HBV Alone	HBV + HCC	ES	*p*
Sex [*n* (%)]	Male	45 (76.3)	39 (97.5)	0.291	0.004
	Female	14 (23.7)	1 (2.5)		
Blood group [*n* (%)]	0	20 (33.9)	15 (37.5)	0.203	0.667
	A	27 (45.8)	17 (42.5)		
	B	10 (16.9)	5 (12.5)		
	AB	2 (3.4)	3 (7.5)		
LT type [*n* (%)]	LDLT	56 (94.9)	37 (92.5)	0.050	0.621
	DDLT	3 (5.1)	3 (7.5)		
Child–Pugh class [*n* (%)]	A	6 (10.2)	17 (45.9)	0.419	<0.001
	B	33 (55.9)	15 (40.5)		
	C	20 (33.9)	5 (13.5)		
Sarcopenia (Based on PMI Cut Off) [*n* (%)]	PreLT	21 (35.6)	12 (30.0)	0.058	0.717
	POD180	28 (47.5)	18 (45.0)	0.024	0.972
	POD365	26 (44.1)	15 (37.5)	0.065	0.658
Outcome [*n* (%)]	Survivors	55 (93.2)	29 (72.5)	0.284	0.005
	Non-survivors	4 (6.8)	11 (27.5)		

Abbreviations: HBV, hepatitis B virus; HCC, hepatocellular carcinoma; ES, effect size; LT, liver transplantation; LDLT, living donor liver transplantation; DDLT, deceased donor liver transplantation; PMI, psoas muscle index; PreLT, pre-liver transplantation; POD180, postoperative day 180; POD365, postoperative day 365.

**Table 3:** Summary of repeated-measures ANOVA results for longitudinal changes in PMA across clinical subgroups.

Between-Subject Factor	Time Effect (*p*)	Partial η^2^ (Time)	Overall Between-Group Effect (*p*)	Time × Factor Interaction (*p*)
HBV Alone vs. HBV + HCC	0.006	0.054	0.359	0.469
Female vs. Male	0.133	0.021	<0.001	0.349
Child–Pugh class (A–C)	0.015	0.046	0.986	0.865
Survivors vs. Non-survivors	0.113	0.023	0.313	0.160

*p* values for time effects and interaction terms are Greenhouse–Geisser corrected. Partial η^2^ values are reported for time effects as measures of effect size.

**Table 4:** Summary of repeated-measures ANOVA results for longitudinal changes in PMI.

Between-Subject Factor	Time Effect (*p*)	Partial η^2^ (Time)	Overall Between-Group Effect (*p*)	Time × Factor Interaction (*p*)
HBV Alone vs. HBV + HCC	0.009	0.051	0.387	0.530
Female vs. Male	0.115	0.023	<0.001	0.265
Child–Pugh class (A–C)	0.017	0.044	0.848	0.840
Survivors vs. Non-survivors	0.117	0.022	0.307	0.137

**Table 5:** Summary of repeated-measures ANOVA results for longitudinal changes in L1–4 vertebral trabecular attenuation.

Between-Subject Factor	Time Effect (*p*)	Partial η^2^ (Time)	Overall Between-Group Effect (*p*)	Time × Factor Interaction (*p*)
HBV Alone vs. HBV + HCC	<0.001	0.443	0.109	0.515
Female vs. Male	<0.001	0.246	0.047	0.102
Child–Pugh class (A–C)	<0.001	0.388	0.100	0.593
Survivors vs. Non-survivors	<0.001	0.374	0.389	0.026

## Data Availability

The data presented in this study are available on request from the corresponding author. The data are not publicly available due to privacy and ethical restrictions.

## Supplementary Materials

The following supporting information can be downloaded at: https://www.mdpi.com/article/10.3390/diagnostics16091340/s1, Table S1: Bivariate comparisons of PMA across clinical subgroups at each time point. Table S2: Bivariate comparisons of PMI between clinical subgroups at each time point. Table S3: Bivariate comparisons of L1–4 vertebral trabecular attenuation according to clinical subgroups at each time point.
