# Supplementary material for: Longitudinal CT-Based Assessment of Muscle and Bone Changes After Liver Transplantation in Hepatitis B Patients with and Without Hepatocellular Carcinoma
Source: Diagnostics (Basel). 2026 Apr 29;16(9):1340. doi: 10.3390/diagnostics16091340 (PMC13163332; doi:10.3390/diagnostics16091340)
Supplement: Supplementary file 1 [file diagnostics-16-01340-s001.zip › diagnostics-4207744-supplementary.pdf]

Supplementary Table S1: Bivariate comparisons of PMA across clinical subgroups at each time point.

Variables	Category	PMA (PreLT)	PMA (POD180)	PMA (POD365)	p (PreLT)	p (POD180)	p (POD365)
Groups	HBV Alone	16.2 ± 5.8	15.5 ± 5.9	15.3 ± 5.5	0.226	0.708	0.342
	HBV+HCC	17.5 ± 4.4	15.9 ± 4.7	16.4 ± 5.5			
Sex	Male	17.8 ± 4.9	16.7 ± 5.1	16.7 ± 5.4	<0.001	<0.001	<0.001
	Female	10.5 ± 3.3	9.5 ± 2.4	10.7 ± 2.4			
Child–Pugh	A	16.9 ± 5.4	15.7 ± 5.0	16.0 ± 4.7	0.967	0.954	0.919
	B	16.6 ± 5.6	15.4 ± 6.1	16.1 ± 5.3			
	C	16.7 ± 4.9	15.8 ± 5.1	15.6 ± 6.0			
Outcome	Survivors	17.0 ± 5.5	15.6 ± 5.7	16.1 ± 5.6	0.236	0.877	0.152
	Non-survivors	15.2 ± 3.9	15.4 ± 3.8	13.9 ± 4.4			

Supplementary Table S2: Bivariate comparisons of PMI between clinical subgroups at each time point.

Variables	Category	PMI (PreLT)	PMI (POD180)	PMI (POD365)	p (PreLT)	p (POD180)	p (POD365)
Groups	HBV Alone	5.5 ± 1.8	5.2 ± 1.8	5.2 ± 1.7	0.227	0.749	0.409
	HBV+HCC	5.9 ± 1.4	5.3 ± 1.6	5.5 ± 1.8			
Sex	Male	5.9 ± 1.5	5.5 ± 1.7	5.5 ± 1.7	<0.001	<0.001	<0.001
	Female	4.1 ± 1.2	3.7 ± 0.9	4.2 ± 0.9			
Child–Pugh	A	5.7 ± 1.6	5.4 ± 1.7	5.5 ± 1.4	0.760	0.771	0.964
	B	5.5 ± 1.6	5.1 ± 1.8	5.4 ± 1.6			
	C	5.7 ± 1.6	5.4 ± 1.7	5.3 ± 2.0			
Outcome	Survivors	5.7 ± 1.7	5.3 ± 1.8	5.4 ± 1.7	0.141	0.961	0.131
	Non-survivors	5.2 ± 1.2	5.2 ± 1.3	4.7 ± 1.4			

Supplementary Table S3: Bivariate comparisons of L1–4 vertebral trabecular attenuation according to clinical subgroups at each time point.

Variables	Category	L1–4 (PreLT)	L1–4 (POD180)	L1–4 (POD365)	p (PreLT)	p (POD180)	p (POD365)
Groups	HBV Alone	190 ± 45	158 ± 43	163 ± 41	0.289	0.060	0.143
	HBV+HCC	181 ± 38	142 ± 38	150 ± 43			
Sex	Male	190 ± 40	153 ± 41	161 ± 40	0.018	0.340	0.032
	Female	162 ± 50	142 ± 49	136 ± 46			
Child–Pugh	A	170 ± 42	140 ± 45	145 ± 44	0.048	0.250	0.195
	B	196 ± 45	157 ± 42	165 ± 44			
	C	184 ± 35	156 ± 36	159 ± 34			
Outcome	Survivors	186 ± 43	153 ± 41	160 ± 41	0.823	0.344	0.097
	Non-survivors	188 ± 41	142 ± 46	141 ± 47			
